# Mental Well-Being Among Emerging Adults With and Without SLD/ADHD During War

**DOI:** 10.3390/ejihpe16100147

**Published:** 2026-09-24

**Authors:** Raaya Alon, Ayelet Harel-Gadassi

**Affiliations:** Special Education, Michlalah Jerusalem College, P.O. Box 16078, Jerusalem 9116002, Israel

**Keywords:** executive functioning, psychological capital, health-promoting daily activities, social support, national crisis

## Abstract

Emerging adulthood is marked by increasing independence and responsibility across educational, occupational, and social domains. During wartime, disruptions to daily routines, security, and perceived control may threaten mental well-being, understood as positive emotional, social, and psychological functioning. These challenges may be particularly salient for individuals with specific learning disabilities (SLD) and/or attention deficit hyperactivity disorder (ADHD), who may experience difficulties in executive functioning and maintaining everyday routines. This study examined differences in mental well-being between emerging adults with and without SLD and/or ADHD and assessed the contributions of executive functioning, health-promoting daily activities, psychological capital, and perceived social support during wartime. Participants were 317 Israeli emerging adults aged 18–28 years. Among the 316 participants with valid diagnostic-group classification, 115 reported SLD and/or ADHD and 201 reported neither condition. Emerging adults with SLD and/or ADHD reported lower mental well-being, greater executive functioning difficulties, and lower engagement in health-promoting daily activities than those without SLD and/or ADHD. Mental well-being was positively associated with psychological capital, health-promoting daily activities, and perceived social support, and negatively associated with executive functioning difficulties. In the hierarchical regression, diagnostic-group status was initially associated with mental well-being but was no longer significant after psychological capital, health-promoting daily activities and executive functioning were entered; perceived social support made a small additional contribution. The final model explained 37.2% of the variance in mental well-being. These findings highlight the relevance of cognitive-functional, psychological, occupational, and social factors to mental well-being during a prolonged national crisis.

## 1. Introduction

The “Iron Swords” war, the name used in Israel for the war that began following the attacks of 7 October 2023, has created an ongoing reality in Israel marked by security threats, uncertainty, exposure to traumatic events, and widespread disruption of daily routines (Dopelt & Houminer-Klepar, 2024; Essa-Hadad et al., 2026; Lipskaya-Velikovsky et al., 2025). These conditions may be especially significant during emerging adulthood, a developmental period generally spanning the late teens through the twenties (approximately ages 18–29), and characterized by identity exploration, increasing independence, entry into higher education or employment, formation of mature interpersonal relationships, and future planning (Arnett et al., 2014). In Israel, this developmental period also intersects with compulsory military service, recent discharge, reserve duty, and close relationships with family members or peers serving in the military. War may therefore affect not only emotional stability but also daily functioning and engagement with central developmental tasks.

Mental well-being is understood in the present study as positive mental health, reflected in emotional, social, and psychological functioning. Rather than focusing only on symptoms of distress, this concept emphasizes positive affect, meaning, competence, belonging, and effective functioning in daily life (Keyes et al., 2008). During wartime, these aspects of well-being may be weakened by ongoing threats, reduced sense of security, loss of routine, diminished personal control, and changes in sleep, study, work, social participation, and health-related habits.

Among emerging adults, those with specific learning disabilities (SLD) and/or attention deficit hyperactivity disorder (ADHD) may face additional challenges. Although SLD and ADHD are often discussed in relation to academic performance, their implications extend beyond learning. They may be associated with difficulties in executive functioning and self-regulation, which can affect organization, time management, task persistence, emotional regulation, and the maintenance of stable routines (Al-Yagon & Toren, 2025; Kosana et al., 2023; Sharabi et al., 2026; Wymbs et al., 2021). These difficulties may become more pronounced during emerging adulthood, when individuals are expected to manage multiple life domains with less external support. In wartime, when environmental demands increase and familiar routines are disrupted, such difficulties may further impact adaptation and mental well-being (Al-Yagon & Toren, 2025; Kosana et al., 2023).

At the same time, diagnostic status alone does not fully explain how emerging adults function or maintain well-being during a crisis. Consistent with the conservation of resources theory, adaptation under stress depends on the availability and preservation of resources across multiple domains (Hobfoll, 1989). Accordingly, the variables examined in the present study can be viewed as complementary resources: executive functioning as a cognitive capacity supporting planning and regulation (Drigas & Karyotaki, 2019; Fombouchet et al., 2024); health-promoting daily activities as a factor reflecting engagement in everyday activities and roles that support participation, meaning, and a sense of control (J. J. Budman et al., 2023; J. R. Budman et al., 2025; Lipskaya-Velikovsky et al., 2025); psychological capital as an internal resource (Luthans et al., 2007); and perceived social support as an external interpersonal resource (Zimet et al., 1988).

Although previous studies have examined the psychological impact of war on students and young adults (Dopelt & Houminer-Klepar, 2024; Essa-Hadad et al., 2026; Lipskaya-Velikovsky et al., 2025; Pavlova et al., 2024), and others have addressed SLD, ADHD, executive functioning, health-promoting daily activities, psychological capital, or social support separately, less is known about how these factors are jointly associated with mental well-being in the context of wartime disruption among emerging adults with and without SLD and/or ADHD. These resources are relevant to emerging adults generally during crisis situations but may be particularly important for those with SLD and/or ADHD, whose pre-existing difficulties in executive functioning, self-regulation, and maintaining routines may increase vulnerability when environmental demands intensify. This knowledge gap is particularly relevant in the Israeli context, where emerging adulthood may intersect with higher education, employment, military service, reserve duty, and exposure to ongoing national threats (Al-Yagon & Toren, 2025; Arnett et al., 2014; Essa-Hadad et al., 2026). Accordingly, the present study compared mental well-being among emerging adults with and without SLD and/or ADHD in Israel during the “Iron Swords” war. It further explored the associations between mental well-being and executive functioning, health-promoting daily activities, psychological capital, and perceived social support, as well as the relative contribution of these variables to mental well-being during wartime.

### 1.1. Mental Well-Being Among Emerging Adults During Wartime

Mental well-being is conceptualized in the present study according to Keyes’s model of positive mental health as a multidimensional construct encompassing emotional, social, and psychological well-being (Keyes et al., 2008). Emotional well-being refers to positive affect and happiness; social well-being to belonging, contribution, and acceptance; and psychological well-being to self-acceptance, personal growth, competence, and effective functioning. This positive-functioning perspective is particularly relevant during prolonged crises, which may undermine routines, social participation, personal control, meaning, and functioning (Seleznova et al., 2023; Essa-Hadad et al., 2026; Lipskaya-Velikovsky et al., 2025).

Emerging adulthood is especially relevant to mental well-being because it involves increasing autonomy and responsibility in education, employment, financial independence, and interpersonal relationships (Tanner & Arnett, 2016). In Israel, this stage may also intersect with military service, reserve duty, and exposure to security threats. War may therefore disrupt both everyday routines and central developmental tasks and may undermine individuals’ sense of control, competence, and meaning. Illustrating this, research has shown that exposure to terror-related stress during emerging adulthood is associated with diminished self-efficacy and self-esteem and increased self-criticism (Schiller et al., 2019). Further, research conducted in Israel following the “Iron Swords” war has documented both lower mental well-being and elevated psychological distress, including depression, anxiety, and stress, among students and emerging adults (Essa-Hadad et al., 2026; Lipskaya-Velikovsky et al., 2025). Residence in conflict zones and increased social media use were associated with higher stress (Dopelt & Houminer-Klepar, 2024), while reserve service was associated with poorer perceived mental health and greater distress (Essa-Hadad et al., 2026). These findings suggest that wartime mental well-being may be shaped not only by direct exposure to risk, but also by disruptions to daily routines, perceived control, and support systems.

### 1.2. Emerging Adults with SLD and/or ADHD

SLD and ADHD are neurodevelopmental conditions that may affect functioning throughout the lifespan. SLD involves persistent difficulties in acquiring and using academic skills, whereas ADHD is characterized by persistent patterns of inattention and/or hyperactivity-impulsivity that interfere with functioning (American Psychiatric Association, 2022). Although often discussed in educational contexts, the implications of these conditions extend into emerging adulthood, when individuals are increasingly expected to juggle education, employment, relationships, daily responsibilities, and independent living. Under these demands, difficulties in self-management, organization, emotional regulation, and maintaining routines may become more evident (Al-Yagon & Toren, 2025; Kosana et al., 2023; Wymbs et al., 2021) and may undermine competence, autonomy, personal control, and social belonging. Accordingly, SLD and/or ADHD may be associated with mental well-being not merely through diagnostic status, but through their impact on everyday functioning and participation.

These difficulties may become more salient during wartime, when emerging adults with SLD and/or ADHD must cope with disrupted routines, changing academic or occupational demands, military or reserve service, and concern for family members. In such circumstances, pre-existing difficulties in executive functioning or emotional regulation may hinder adaptation (Al-Yagon & Toren, 2025; Kosana et al., 2023). At the same time, the mere presence of a diagnosis does not fully account for levels of mental well-being. Mental well-being among emerging adults with SLD and/or ADHD may also be associated with internal resources, social support, and the ability to maintain meaningful daily activities and routines. Therefore, a broader perspective is required, integrating diagnostic characteristics with functional, psychological, and social factors.

### 1.3. Executive Functioning Among Emerging Adults

Executive functions constitute a set of regulatory, monitoring, and control processes that enable individuals to direct their thoughts, emotions, and behaviors toward goal attainment. These functions include attentional control, cognitive flexibility, emotional regulation, working memory, response inhibition, planning, organization, and problem-solving (Drigas & Karyotaki, 2019; Fombouchet et al., 2024). Among emerging adults, these functions are particularly critical because they support the independent management of educational, occupational, social, and daily-life demands (Cushman et al., 2022; Wymbs et al., 2021).

During wartime, executive functioning may become especially important because individuals must manage changing information, competing demands, disrupted routines, and emotional stress. At the same time, acute stress may impair working memory, cognitive flexibility, and cognitive inhibition (Shields et al., 2016), whereas stronger attentional control and working memory have been associated with lower stress responses (Grimm et al., 2021). Executive functioning difficulties have also been linked to poorer mental health-related quality of life among college students and adults with ADHD (Cushman et al., 2022; Zhang et al., 2021). These findings suggest that executive functioning may support adaptation by helping individuals maintain routines, manage responsibilities, regulate emotion, and preserve a sense of competence and control under changing conditions.

Executive functioning challenges may be particularly consequential for emerging adults with SLD and/or ADHD, who often experience difficulties in time management, task organization, working memory, and monitoring ongoing demands (Al-Yagon & Toren, 2025; Wymbs et al., 2021). Because executive functions support goal-directed behavior, perceived competence, emotion regulation, and continuity of daily functioning, they may represent an important cognitive-functional correlate of mental well-being during wartime.

### 1.4. Health-Promoting Daily Activities as a Resource

Health-promoting daily activities refer here to engagement in everyday activities and roles that contribute to the fulfillment of physical, psychological, and social needs and may support health, meaning, identity, belonging, and psychological resilience (J. R. Budman et al., 2025). Engagement in activities that address physical, psychological, and social needs has been associated with better psychological health and quality of life (J. J. Budman et al., 2023). During wartime, maintaining meaningful and balanced daily activities may take on added relevance. Students during the “Iron Swords” war reported poorer sleep, reduced physical activity, and less healthy eating, while occupational balance and meaning were associated with better mental health (Essa-Hadad et al., 2026; Lipskaya-Velikovsky et al., 2025). Thus, engagement in health-promoting daily activities may serve as a resource for maintaining mental well-being.

Executive functioning supports the initiation, planning, organization, and persistence required for everyday activities (Drigas & Karyotaki, 2019; Wymbs et al., 2021). When executive difficulties are present, such as with SLD and/or ADHD, maintaining routines and meeting daily demands may become more difficult, particularly when wartime disruption is already high. Examining health-promoting daily activities alongside executive functioning may therefore help clarify whether each is independently associated with mental well-being.

### 1.5. Psychological Capital as an Internal Resource

Psychological capital is a positive psychological resource comprising self-efficacy, hope, optimism, and resilience (Luthans et al., 2007; Mamed et al., 2025). These components reflect confidence in coping, goal-directed thinking, positive expectations, and the capacity to recover from adversity.

In wartime contexts, psychological capital may serve as an important internal resource. Among internally displaced people exposed to war, higher psychological capital was associated with lower mental distress and post-traumatic stress symptoms, with self-efficacy emerging as its strongest predictive component (Mamed et al., 2025). Personal resources, including hope, optimism, and resilience, have been associated with subjective well-being among emerging adults living under conditions of war-related threat (Pavlova et al., 2024). In the Israeli context, the importance of resilience-related resources, such as sense of coherence and ego resilience, has been emphasized in adapting to demanding environments such as military service (Al-Yagon & Toren, 2025).

### 1.6. Perceived Social Support as an External Resource

Perceived social support refers to the subjective appraisal that emotional and instrumental support is available from family, friends, and significant others (Zimet et al., 1988). Such support remains important during emerging adulthood, despite increasing independence and changing relationships with family members and other significant figures (Al-Yagon & Toren, 2025; Tanner & Arnett, 2016).

During wartime, social support may provide security, belonging, emotional validation, practical assistance, and continuity (Lipskaya-Velikovsky et al., 2025). Family cohesion, supportive authority figures, peer relationships, and community belonging have been linked to resilience, social adaptation, or mental health in demanding contexts (Al-Yagon & Toren, 2025; Cohen et al., 2024; Lipskaya-Velikovsky et al., 2025). These support systems may become especially important when educational, occupational, and social frameworks are disrupted.

Amongst emerging adults with SLD and/or ADHD, social support plays an important role. For college students with ADHD symptoms, a larger social support network buffered the association between ADHD symptoms and functional impairment (Reeble et al., 2024). Difficulties in executive functioning, emotional regulation, and managing life demands may also increase the need for emotional support, guidance, and practical assistance (Cohen et al., 2024). Social support may therefore complement internal and functional resources in understanding mental well-being.

### 1.7. The Current Study

The above-reviewed literature suggests that mental well-being among emerging adults during wartime is associated with developmental characteristics, war-related stress, executive functioning, health-promoting daily activities, psychological capital, and social support. While emerging adults with SLD and/or ADHD may face additional difficulties related to executive functioning and emotional regulation, executive functioning, health-promoting daily activities, psychological capital, and perceived social support may also be relevant to mental well-being; however, their combined associations have yet to be examined within a single framework, particularly in the context of the “Iron Swords” war. Given the limited evidence regarding the combined role of these factors during wartime, the present study was exploratory in nature, and no a priori hypotheses were formulated. The following research questions emerged:

1. Are there differences between emerging adults with and without SLD and/or ADHD in mental well-being and the key study variables?
2. What are the associations between mental well-being and executive functioning, health-promoting daily activities, psychological capital, and perceived social support?
3. What is the relative contribution of executive functioning, health-promoting daily activities, psychological capital, and perceived social support to mental well-being among emerging adults with and without SLD and/or ADHD during wartime?

An integrated examination guided by these questions may contribute to a deeper understanding of the factors associated with mental well-being among emerging adults and identify domains for further longitudinal and intervention research.

## 2. Methods

### 2.1. Participants

The sample consisted of 317 young adults aged 18–28 years (*M* = 21.61, *SD* = 2.44). The sample included 277 women (87.4%) and 39 men (12.3%); gender was missing for one participant. Participants were categorized according to their self-reported diagnostic status. Valid group classification was available for 316 participants. In total, 201 participants were classified as having no SLD and/or ADHD, and 115 participants were classified as having SLD and/or ADHD. Within the SLD/ADHD group, 29 participants reported SLD only, 23 reported ADHD only, and 59 reported co-occurring SLD and ADHD. Four additional participants reported ADHD but did not provide information regarding SLD status; therefore, they were included in the broader SLD/ADHD group but were not included in the subtype breakdown. Descriptively, the SLD-only subgroup (*n* = 29) had mean scores of 3.53 (*SD* = 0.48) for mental well-being, 2.31 (*SD* = 0.56) for psychological capital, 57.21 (*SD* = 7.98) for GEC, 3.08 (*SD* = 0.59) for health-promoting daily activities, and 3.69 (*SD* = 1.08) for social support. Corresponding values were 3.58 (*SD* = 0.54), 2.28 (*SD* = 0.57), 61.83 (*SD* = 9.22), 3.25 (*SD* = 0.65), and 3.63 (*SD* = 1.11) for the ADHD-only subgroup (*n* = 23), and 3.60 (*SD* = 0.60), 2.42 (*SD* = 0.64), 67.54 (*SD* = 11.93), 3.13 (*SD* = 0.73), and 3.72 (*SD* = 1.18) for the co-occurring SLD/ADHD subgroup (*n* = 59), respectively. For the main analyses, participants with SLD only, ADHD only, co-occurring SLD and ADHD, or a valid report of ADHD with missing SLD subtype information were combined into a single SLD/ADHD group and compared with participants without SLD/ADHD. This grouping reflected the primary aim of comparing emerging adults reporting SLD and/or ADHD with those reporting neither condition, rather than comparing the diagnostic subgroups with one another. The relatively small and unequal sizes of the diagnostic subgroups supported retaining the broader SLD/ADHD grouping for the primary analyses; however, a subgroup-specific sensitivity analysis was conducted to examine the robustness of the findings. Among participants with available data for each variable, 195 (62.3%) were single, 102 (32.6%) were married, and 16 (5.1%) reported another marital status. Regarding religiosity, 204 (66.9%) identified as religious, 87 (28.3%) as ultra-Orthodox, 14 (4.6%) as traditional. Regarding education, 92 participants (29.4%) had completed 12 years of schooling and were enrolled in non-degree vocational studies, 171 (54.6%) held or were pursuing a bachelor’s degree, and 50 (16.0%) held or were pursuing a master’s degree or higher. Sixteen participants (5.1%) served in the military reserves during the war, and 164 (52.6%) reported that a first-degree relative served. Twenty participants (6.4%) reported being directly harmed during the war, and 19 (6.1%) reported that a first-degree relative had been harmed. Participants were eligible to participate if they were 18 to 28 years of age, lived in Israel during the Iron Swords War, were able to complete an online questionnaire, and provided informed consent. Participants were excluded from the analytic sample if they were outside the designated age range, did not provide informed consent, or submitted incomplete responses that prevented classification into the study groups.

### 2.2. Measures

#### 2.2.1. Demographic Questionnaire

Participants completed a demographic questionnaire. The questionnaire included items referring to participants’ general background characteristics, including age, gender, marital status, place of residence, level of religiosity, level of education, employment status, and previous official diagnosis of an SLD and/or ADHD. In addition, participants were asked whether they or a first-degree family member had served in the military during the war and whether they or a first-degree family member had been directly harmed during the war.

#### 2.2.2. Mental Well-Being

Mental well-being was assessed using the Mental Health Continuum–Short Form—MHC-SF—based on Keyes et al. (2008). The questionnaire includes 14 items assessing positive mental health during the past month. The MHC-SF measures three dimensions of well-being. Emotional well-being is assessed by three items, for example: “I felt satisfied with my life.” Social well-being is assessed by five items, for example: “I felt that I had something important to contribute to society.” Psychological well-being is assessed by six items, for example: “I felt that I liked most parts of my personality.” In the version administered in the present study, participants rated the extent to which each statement represented their feelings during the past month on a five-point scale: 1 (to a very small extent), 2 (to a small extent), 3 (to a moderate extent), 4 (to a great extent), and 5 (to a very great extent). The 14 items were averaged to create a total mental well-being score, with higher scores indicating higher levels of mental well-being. In the present study, internal consistency for the total mental well-being scale was high, with Cronbach’s α = 0.90. Internal consistency for the subscales was α = 0.75 for emotional well-being, α = 0.79 for social well-being, and α = 0.84 for psychological well-being.

#### 2.2.3. Psychological Capital

Psychological capital was assessed using the 12-item Psychological Capital Questionnaire developed by Luthans et al. (2007). Items were rated on a 5-point Likert scale and coded so that higher scores indicated greater psychological capital. The questionnaire assesses four components, with three items representing each component: self-efficacy (e.g., “I feel confident analyzing a long-term problem to find a solution”), optimism (e.g., “I am optimistic about what will happen to me in the future regarding work”), hope (e.g., “I can think of many ways to reach my current work goals”), and resilience (e.g., “I usually take stressful things at work in stride”). A general psychological capital score was calculated as the mean of the 12 items. In the present study, internal consistency for the overall psychological capital scale was good with Cronbach’s α = 0.87. Internal consistency for the subscales was α = 0.68 for self-efficacy, α = 0.74 for optimism, α = 0.68 for hope, and α = 0.73 for resilience.

#### 2.2.4. Perceived Social Support

Perceived social support was assessed using the Multidimensional Scale of Perceived Social Support—MSPSS—developed by Zimet et al. (1988). The scale assesses individuals’ subjective perception of available social support from three sources: family, friends, and significant others. The questionnaire includes 12 items, with four items for each source of support. Items were rated on a 5-point Likert-type scale ranging from 1 (to a very small extent) to 5 (to a very great extent), with higher scores indicating greater perceived social support. In the present study, the Hebrew version of the questionnaire translated by Statman (1995) was used. Previous studies have reported high reliability for the questionnaire in both English and Hebrew. In the Hebrew version, Statman (1995) reported high internal consistency for the family support subscale, α = 0.93, the friends support subscale, α = 0.91, and the significant others support subscale, α = 0.91. In the present study, internal consistency for the total perceived social support scale was high, Cronbach’s α = 0.96. Internal consistency for the subscales was α = 0.94 for family support, α = 0.94 for friends support, and α = 0.93 for significant others support.

#### 2.2.5. Executive Functioning

Executive functioning was assessed using the Behavior Rating Inventory of Executive Function–Adult Version (BRIEF-A; Roth et al., 2005). BRIEF-A is a standardized rating scale designed to assess manifestations of executive functioning in adults’ everyday lives. The questionnaire includes 75 items rated on a 3-point Likert-type scale: 1 = “never,” 2 = “sometimes,” and 3 = “often.” Participants were asked to indicate how often each behavior occurred in their daily life. BRIEF-A includes nine clinical scales assessing different aspects of executive functioning: inhibit, shift, emotional control, self-monitor, initiate, working memory, plan/organize, task monitor, and organization of materials. These scales are combined into two broader indexes: the Behavioral Regulation Index—BRI—and the Metacognition Index—MI. Together, these indexes form the Global Executive Composite—GEC, which represents an overall measure of executive functioning difficulties. Higher scores indicate greater executive functioning difficulties.

In the present study, standardized GEC T-scores were used as the primary measure of executive functioning, with higher T-scores indicating greater executive-functioning difficulties. Internal consistency for the GEC was high, Cronbach’s α = 0.96. The BRIEF-A Negativity, Infrequency, and Inconsistency validity indices were calculated from the available item-level responses. Protocols were flagged at scores of ≥6 for Negativity, ≥3 for Infrequency, or ≥8 for Inconsistency. Thirty participants were flagged on at least one validity index. The original BRIEF-A response coding of 1 = never, 2 = sometimes, and 3 = often was retained; responses were not recoded to a 0–2 format before standardized GEC T-scores were derived.

#### 2.2.6. Health-Promoting Daily Activities

Health-promoting daily activities were assessed using seven participation items from the Hebrew version of the Experiencing Day-to-Day Life Questionnaire (EDLQ; J. R. Budman et al., 2025). The Hebrew version was obtained directly from the instrument developer and had previously been used in an Israeli sample of mothers of children with ADHD (J. J. Budman et al., 2023); therefore, no translation was required for the present study. The EDLQ is based on the assumption that engagement in daily activities contributes to the fulfillment of basic physical, psychological, and social needs. In the present study, participants rated the extent to which they had engaged during the previous month in activities across seven domains: caring for physical needs, including medical care, physical activity, and healthy nutrition; financial security, including financial management and work; spirituality, including connection to religious or non-religious values and beliefs; pleasure, joy, and enjoyment; interpersonal relationships and social connections; social and community contribution; and the development and realization of personal abilities. Each item was rated on a 5-point Likert-type scale ranging from 1 (not at all) to 5 (to a very great extent). Because each domain was represented by a single item and the present study focused on overall engagement across health-promoting domains rather than domain-specific patterns, the seven items were averaged to create a single composite score. Higher scores indicated greater overall engagement in health-promoting daily activities. Thus, the composite score should be interpreted as a measure of overall engagement in health-promoting daily activities rather than general day-to-day functioning. In the present study, internal consistency for the seven-item composite was Cronbach’s α = 0.69.

### 2.3. Procedure

The study was approved by Human Research Ethics Committee of Michlalah Jerusalem College, approval number 018. Data were collected between January and April 2024, during the Iron Swords War in Israel. Participants were recruited by 15 research assistants, who were themselves emerging adults and received standardized instructions regarding recruitment procedures and participant eligibility. Convenience and snowball sampling were used. The research assistants distributed a standardized invitation containing a brief description of the study, the eligibility criteria, and a link to the anonymous online questionnaire. The invitation was initially sent to potentially eligible individuals within the assistants’ personal and social networks, who were also invited to forward it to other emerging adults. The questionnaire link was additionally distributed through WhatsApp groups, Facebook groups, and email lists. The research assistants did not determine or record participants’ diagnostic status during recruitment; SLD and ADHD status was reported by participants within the questionnaire. Participation was voluntary, and no identifying information was collected by the research assistants. Before completing the questionnaires, participants received an informed consent form that included information about the purpose of the study, the estimated time required to complete the questionnaires, the voluntary nature of participation, anonymity, and confidentiality of the data collected. Participants were informed that they could withdraw from the study at any stage. After providing informed consent, participants completed the questionnaires online and anonymously. Completion of the questionnaire battery took approximately 15 min. Because data were collected during wartime and included questions related to mental well-being and exposure to war-related events, participants were provided with contact details and were informed that they could contact the research team if needed.

### 2.4. Data Analysis

Data were analyzed using IBM SPSS Statistics, version 29. Descriptive statistics were calculated for demographic variables and for all study variables, including means, standard deviations, frequencies, and internal consistency coefficients. The dataset included 317 respondents. Composite scores for mental well-being, psychological capital, executive functioning, health-promoting daily activities, and perceived social support were available for all participants. Group status was missing for one respondent; therefore, group comparisons were conducted among 316 participants with valid group classification. Correlation analyses were conducted with the full sample of 317 participants. Given the wartime context of the study, war-related exposure variables, including reserve military service and direct or familial war-related harm, were examined. A sensitivity analysis was additionally conducted including these variables as covariates to assess the robustness of the primary regression findings. The hierarchical regression analysis was conducted with 315 participants, because one participant had missing group status and one participant had missing gender. No imputation was conducted; analyses were based on available valid cases. Therefore, valid sample sizes are reported for each analysis. Prior to the inferential analyses, the data were examined for normality, outliers, and multicollinearity. No substantial violations were identified.

Independent samples *t*-tests were conducted to examine differences between participants with and without SLD/ADHD in the study variables: mental well-being, psychological capital, executive functioning, health-promoting daily activities, and perceived social support. Pearson correlation analyses were conducted to examine associations among mental well-being, psychological capital, executive functioning, health-promoting daily activities, and perceived social support. Potential demographic and contextual differences between participants with and without SLD/ADHD were examined using an independent-samples *t*-test for age and chi-square tests for categorical variables. Based on these group comparisons, an additional sensitivity analysis examined whether the primary regression findings remained robust after controlling for education and religiosity, in addition to gender, which was already included in the primary model. An additional sensitivity analysis repeated the hierarchical regression after excluding cases flagged by the BRIEF-A validity indices. A further sensitivity analysis replaced the broad SLD/ADHD grouping with separate indicators for SLD only, ADHD only, and co-occurring SLD/ADHD, with participants reporting neither condition as the reference group. Participants with incomplete diagnostic-subgroup information were excluded from this analysis Finally, a hierarchical regression analysis was conducted to examine the relative contribution of demographic, diagnostic, functional, executive, psychological, and social variables to the prediction of mental well-being. In the first step, gender and group status were entered. Gender was coded as 0 = female and 1 = male. Group status was dummy-coded, with participants without SLD/ADHD coded as 0 and participants with SLD/ADHD coded as 1. In the second step, health-promoting daily activities, GEC, and psychological capital were added. In the third step, perceived social support was added. The level of statistical significance was set at *p* < 0.05.

## 3. Results

### 3.1. Differences in Study Variables Between Individuals With/Without SLD/ADHD

Results of the independent samples *t*-tests examining differences between emerging adults with and without SLD/ADHD in terms of the study variables are presented in Table 1. Significant group differences were found in mental well-being, GEC, and health-promoting daily activities. Specifically, well-being and health-promoting daily activities were significantly higher among emerging adults without SLD/ADHD than among those with SLD/ADHD, while GEC was significantly higher among participants with SLD/ADHD than among those without SLD/ADHD. No other significant differences were detected between the groups.

Additional comparisons of demographic and contextual characteristics indicated significant group differences in gender, χ^2^(1) = 8.91, *p* = 0.003, education, χ^2^(2) = 11.50, *p* = 0.003, and religiosity, χ^2^(2) = 10.91, *p* = 0.004. The SLD/ADHD group included a higher proportion of men and a higher proportion of participants in the non-degree vocational education and traditional categories than the group without SLD/ADHD. The SLD/ADHD group also included a lower proportion of ultra-Orthodox participants. No significant group differences were found in age, *t*(314) = −0.18, *p* = 0.855, marital status, χ^2^(2) = 1.97, *p* = 0.374, reserve military service, χ^2^(1) = 0.83, *p* = 0.363, or direct/familial war-related harm, χ^2^(1) = 3.18, *p* = 0.075.

### 3.2. Correlations

Pearson correlations among the study variables are presented in Table 2. Mental well-being was positively correlated with psychological capital, health-promoting daily activities, and perceived social support, indicating that higher levels of these resources were associated with higher mental well-being. GEC was negatively correlated with mental well-being, indicating that greater executive-functioning difficulties were associated with lower mental well-being. GEC was also negatively correlated with psychological capital and health-promoting daily activities. In addition, psychological capital was positively correlated with health-promoting daily activities, and health-promoting daily activities were positively correlated with perceived social support. Intercorrelations among the explanatory variables were low to moderate, consistent with the absence of substantial multicollinearity.

### 3.3. Predicting Mental Well-Being

Results of the hierarchical regression analysis are presented in Table 3. An examination of the table reveals that Step 1, which included gender and diagnostic status (with/without SLD/ADHD) was statistically significant, *F*(2, 312) = 4.73, *p* = 0.009, with only SLD/ADHD status contributing significantly to the explained variance. Step 2, with the addition of psychological capital, GEC, and greater engagement in health-promoting daily activities was also significant, *F*(5, 309) = 34.94, *p* < 0.001, and with these variables, gender and SLD/ADHD status were no longer significant. Step 3 added social support, and was also statistically significant, *F*(6, 308) = 30.47, *p* < 0.001. In other words, higher levels of psychological capital, greater engagement in health-promoting daily activities, and social support were associated with higher levels of mental well-being, while higher GEC (i.e., lower executive functioning) was associated with lower well-being. A sensitivity analysis including reserve military service and direct or familial war-related harm as covariates yielded a comparable pattern of findings. An additional sensitivity analysis controlling for education and religiosity, in addition to gender already included in the primary model, yielded the same overall pattern of findings, with psychological capital, executive functioning, health-promoting daily activities, and perceived social support remaining significantly associated with mental well-being, whereas diagnostic-group status remained non-significant. When the regression analysis was repeated after excluding the 30 participants flagged on at least one BRIEF-A validity index, the resulting analytic sample included 285 participants. The model remained statistically significant, *F*(6, 278) = 29.43, *p* < 0.001, R^2^ = 0.388. Psychological capital (β = 0.328, *p* < 0.001), GEC (β = −0.121, *p* = 0.021), health-promoting daily activities (β = 0.298, *p* < 0.001), and perceived social support (β = 0.116, *p* = 0.016) remained significantly associated with mental well-being, whereas diagnostic-group status remained non-significant (β = −0.071, *p* = 0.160). Thus, the substantive pattern of findings was unchanged. The subgroup-specific sensitivity analysis included 311 participants with complete diagnostic-subgroup and regression data. The final model was statistically significant, *F*(8, 302) = 23.45, *p* < 0.001, R^2^ = 0.383. With participants reporting neither SLD nor ADHD as the reference group, none of the diagnostic subgroup indicators was significantly associated with mental well-being: SLD only (β = −0.056, *p* = 0.239), ADHD only (β = −0.046, *p* = 0.323), or co-occurring SLD/ADHD (β = 0.031, *p* = 0.552). Psychological capital, GEC, health-promoting daily activities, and perceived social support remained significant. Thus, modeling the diagnostic subgroups separately did not alter the substantive pattern of findings.

Taken together, the findings directly address the three research questions. Regarding RQ1, participants with self-reported SLD/ADHD reported lower mental well-being, greater executive-functioning difficulties, and lower engagement in health-promoting daily activities, whereas psychological capital and perceived social support did not differ significantly between the groups. Regarding RQ2, mental well-being was positively associated with psychological capital, health-promoting daily activities, and perceived social support, and negatively associated with executive-functioning difficulties. Regarding RQ3, psychological capital, health-promoting daily activities, executive-functioning difficulties, and perceived social support were independently associated with mental well-being in the final regression model, whereas diagnostic-group status and gender were not.

## 4. Discussion

The present study examined mental well-being among emerging adults in Israel during the “Iron Swords” war, with particular attention to differences between individuals with and without SLD and/or ADHD and to the role of cognitive-functional, occupational, psychological, and social resources. Taken together, the findings suggest that mental well-being during wartime cannot be understood through diagnostic status alone. Rather, it appears to be associated with emerging adults’ capacity to regulate cognitive and emotional demands, maintain meaningful daily routines that include health-promoting activities, draw on internal psychological resources, and perceive support from their social environment. Viewing mental well-being as reflecting the combined contribution of cognitive-functional, occupational, psychological, and social resources is particularly relevant during emerging adulthood, a developmental period marked by transition, increasing independence, and heightened sensitivity to uncertainty and instability (Arnett et al., 2014). In the context of a national crisis, mental well-being may be associated not only with the level of stress to which emerging adults are exposed, but also with their ability to maintain routines, function in daily life, and feel some control over their circumstances.

### 4.1. Group Differences

Group comparisons provide an important first step in understanding mental well-being in this wartime sample. Emerging adults with SLD and/or ADHD reported lower mental well-being, greater executive functioning difficulties, and lower scores on health-promoting daily activities than their peers without SLD and/or ADHD. These group differences should be interpreted as associations with self-reported SLD/ADHD status rather than differences between clinically verified diagnostic groups. These findings are consistent with previous work indicating that the implications of SLD and ADHD extend beyond academic performance to difficulties in self-management, emotional regulation, organization, persistence, and adaptation to demanding environments (Al-Yagon & Toren, 2025; Kosana et al., 2023). By contrast, no significant group differences were found in psychological capital or perceived social support, suggesting that these resources were similarly distributed across the two groups. Taken together, the group differences were most evident in mental well-being, executive functioning, and health-promoting daily activities. In the regression model, diagnostic-group status was no longer significantly associated with mental well-being after executive functioning, health-promoting daily activities, and psychological capital were entered. Given the cross-sectional design, this attenuation should not be interpreted as evidence of mediation or causal directionality. A comparable pattern was reported by Sharabi et al. (2026), who found that academic self-efficacy and self-regulated learning mediated the association between SLD/ADHD status and academic achievement among higher education students. Although their outcome was academic achievement rather than mental well-being, the present findings are consistent with an association between psychological and self-regulatory factors and outcomes beyond diagnostic status, without establishing mediation or causal directionality.

### 4.2. Resources Associated with Mental Well-Being

The regression findings highlight complementary domains associated with mental well-being: psychological resources, engagement in health-promoting daily activities, executive functioning, and social resources. Psychological capital, including hope, optimism, resilience, and self-efficacy, represents a psychological resource that may help emerging adults view stressful situations as manageable and feel more capable of coping with uncertainty. Previous studies have similarly linked these kinds of personal resources with better well-being and lower distress in war-related or highly stressful contexts (Mamed et al., 2025; Pavlova et al., 2024). Health-promoting daily activities, by contrast, represent engagement in everyday activities and roles across multiple life domains. Greater engagement in these activities may help individuals preserve meaningful routines, participate in everyday roles, and experience balance and continuity in daily life. This is consistent with occupational science and occupational therapy perspectives, which emphasize daily activity as a source of health, meaning, identity, and psychological functioning (J. J. Budman et al., 2023; J. R. Budman et al., 2025). Evidence from the COVID-19 crisis similarly indicates that disruptions to daily routines are associated with greater symptoms of depression, anxiety, and psychological distress (Liu et al., 2024). During wartime, when sleep, study, work, social participation, and health-related habits may be disrupted, the ability to maintain meaningful daily activities may be particularly relevant to mental well-being (Lipskaya-Velikovsky et al., 2025). Together, these findings suggest that mental well-being during a crisis is associated not only with psychological resources, but also with the ability to maintain everyday routines and roles.

Executive functioning difficulties also made a unique negative contribution to the explanation of the variance in mental well-being after diagnostic-group status, psychological capital, health-promoting daily activities, and perceived social support were taken into account. Executive functioning supports planning, organization, cognitive flexibility, emotion regulation, and the maintenance of daily routines under changing conditions (Drigas & Karyotaki, 2019; Fombouchet et al., 2024). These capacities may be particularly relevant during wartime, when individuals must adjust plans, process changing information, and respond flexibly to uncertainty. This finding is also consistent with evidence that SLD and ADHD are often associated with difficulties in organization, time management, persistence, and adaptation to demanding environments (Al-Yagon & Toren, 2025; Wymbs et al., 2021).

Perceived social support added a small but significant contribution to the explanation of the variance in mental well-being in the final step of the regression model. This suggests that support from family, friends, and significant others remains important during wartime, but should be considered alongside individuals’ internal and functional resources. Social support may provide emotional security, practical assistance, validation, and a sense of belonging, all of which may be particularly important during periods of uncertainty (Al-Yagon & Toren, 2025; Zimet et al., 1988). At the same time, the relatively small additional contribution of social support may indicate that support alone is not sufficient when emerging adults struggle to organize daily life, regulate demands, or maintain meaningful routines. This interpretation is strengthened by the finding that participants with and without SLD and/or ADHD did not differ significantly in perceived social support, raising the possibility that the effectiveness of support may depend not only on the amount of support perceived, but also on the extent to which it facilitates coping and daily functioning. For emerging adults with SLD and/or ADHD, social support may encompass both emotional and practical forms of assistance. Further research is needed to examine which forms of support are most closely associated with mental well-being during crisis.

### 4.3. Limitations and Future Research

The present study has several strengths, including its examination of emerging adults during an ongoing national crisis and its integration of cognitive-functional, occupational, psychological, and social resources within a single analytic framework. Nevertheless, several limitations should be acknowledged. First, the study employed a cross-sectional design, and causal conclusions cannot be drawn regarding the relationships between executive functioning, health-promoting daily activities, psychological capital, perceived social support, and mental well-being. Although the findings indicate meaningful associations among these variables, they do not allow conclusions about directionality. For example, stronger psychological capital and greater engagement in health-promoting daily activities may support mental well-being, but higher mental well-being may also enable individuals to function more effectively, maintain routines, and perceive their resources more positively. Accordingly, these variables should not be interpreted as established protective mechanisms, mediators, or intervention targets. Future longitudinal studies are therefore needed to examine changes in mental well-being over time during prolonged crisis and to clarify whether cognitive-functional, occupational, psychological, and social resources predict later adjustment.

Second, the data were based on self-report questionnaires, including participants’ self-reported SLD and/or ADHD status. Self-report measures are appropriate for capturing subjective experiences such as perceived support, psychological resources, daily functioning, and mental well-being. However, they may also be influenced by reporting bias, current emotional state, or differences in self-awareness. Future studies could strengthen the findings by including clinical or educational documentation of SLD and/or ADHD, behavioral measures of executive functioning, or reports from additional sources. Several measurement-related limitations should also be noted. The MHC-SF was administered using a five-point response format rather than the original six-category format, which may limit direct comparability with studies using the standard scoring procedure. In addition, although sensitivity analyses excluding participants flagged by the BRIEF-A validity indices yielded the same substantive pattern of findings, some uncertainty related to self-report response quality remains. The health-promoting daily activities composite also showed modest internal consistency (α = 0.69) and should therefore be interpreted as a broad indicator of engagement across these domains.

Third, the study relied on convenience and snowball sampling and included a high proportion of women. These characteristics may limit the representativeness of the sample and the generalizability of the findings to the broader population of emerging adults, particularly men and individuals not reached through the recruitment networks. The sample was also predominantly religious or ultra-Orthodox, which may further limit generalizability to more religiously diverse or secular populations. Although sensitivity analyses controlling for education and religiosity yielded the same overall pattern of findings, demographic differences between the groups and other unmeasured contextual factors may still have contributed to the observed associations. Future research should examine whether similar patterns are found among men and women and whether gender moderates the associations between executive functioning, health-promoting daily activities, psychological capital, social support, and mental well-being during wartime. Future research may also examine autonomy as a potentially relevant factor in the mental well-being of emerging adults.

Fourth, participants with SLD only, ADHD only, and co-occurring SLD and ADHD were combined into a single SLD/ADHD group for the main analyses. This approach enabled comparisons between emerging adults with and without neurodevelopmental difficulties, but it may have obscured meaningful differences between these subgroups. However, the subgroup-specific sensitivity analysis yielded the same substantive pattern as the primary analysis. Future studies with larger samples should examine SLD, ADHD, and co-occurring SLD/ADHD separately in order to identify distinct profiles of vulnerability, functioning, and coping resources.

Finally, the study was conducted in the specific context of the “Iron Swords” war in Israel. This context provides an important opportunity to examine mental well-being during an ongoing national crisis, but the findings should be interpreted with attention to the cultural, educational, social, and military realities of Israeli emerging adulthood. Future research should examine whether similar patterns emerge in other countries, crisis contexts, and populations of emerging adults. However, because the study did not include a pre-war comparison or baseline and assessed war exposure using a limited set of indicators, the findings should be interpreted as associations observed within a wartime context rather than as effects specifically attributable to the war.

### 4.4. Implications and Conclusions

From a theoretical perspective, the study extends previous work on risk and resilience among young adults with SLD and/or ADHD in demanding contexts (Al-Yagon & Toren, 2025) by showing how cognitive-functional, occupational, psychological, and social factors are jointly associated with mental well-being under prolonged uncertainty. The findings also identify several domains that may warrant further investigation in supportive interventions for emerging adults with SLD and/or ADHD during periods of crisis. Future intervention studies could examine whether approaches addressing executive functioning, routine management, engagement in health-promoting daily activities, psychological capital, and social support contribute to improved mental well-being. In higher education settings, this direction is consistent with Sharabi et al. (2026), who emphasized strengthening self-regulated learning, academic self-efficacy, time management, organization, and planning alongside the academic accommodations typically provided to students with SLD/ADHD.

The present study highlights the importance of understanding mental well-being among emerging adults during wartime through an integrative lens. The findings shift the focus from diagnosis of SLD and/or ADHD as a fixed risk factor to the everyday factors associated with vulnerability and resilience during crisis. Taken together, the findings identify executive functioning, health-promoting daily activities, psychological capital, and perceived social support as relevant domains for understanding mental well-being beyond diagnostic status. These domains warrant further longitudinal and intervention research during periods of prolonged uncertainty.

## Figures and Tables

**Table 1:** Differences Between Emerging Adults With and Without SLD/ADHD.

	With SLD/ADHD		Without SLD/ADHD		*t*(314)	*p*
Variables	*M*	*SD*	*M*	*SD*		
Well-being	3.57	0.55	3.75	0.57	2.74	0.007
PsyCap	2.36	0.60	2.25	0.57	−1.60	0.111
GEC	63.58	11.19	54.87	10.85	−6.79	<0.001
Health-promoting daily activities	3.14	0.68	3.30	0.63	2.04	0.042
Support	3.68	1.12	3.85	1.02	1.40	0.163

*Note.* With SLD/ADHD *n* = 115, without SLD/ADHD *n* = 201. PsyCap = psychological capital. GEC = Global Executive Composite.

**Table 2:** Correlations Among Study Variables.

	1	2	3	4	5
1. Well-being	--------				
2. PsyCap	0.485 ***	---------			
3. GEC	−0.308 ***	−0.295 ***	--------		
4. Health-promoting daily activities	0.486 ***	0.378 ***	−0.217 ***	-------	
5. Social support	0.208 ***	0.078	−0.105	0.184 **	------

*Note.* PsyCap = psychological capital. GEC = Global Executive Composite. ** p < 0.01, *** p < 0.001.

**Table 3:** Hierarchical Regression Predicting Mental Well-Being.

Step	Variables	B	SE (B)	β	R^2^	ΔR^2^
1					0.029 **	0.029 **
	Gender	0.127	0.098	0.074		
	SLD/ADHD	−0.199	0.067	−0.169 **		
2					0.361 ***	0.332 ***
	Gender	−0.016	0.081	−0.009		
	SLD/ADHD	−0.048	0.059	−0.040		
	PsyCap	0.298	0.050	0.307 ***		
	GEC	−0.006	0.002	−0.128 *		
	Health-promoting daily activities	0.298	0.044	0.341 ***		
3					0.372 ***	0.011 *
	Gender	−0.009	0.080	−0.005		
	SLD/ADHD	−0.044	0.058	−0.037		
	PsyCap	0.299	0.049	0.308 ***		
	GEC	−0.006	0.002	−0.121 *		
	Health-promoting daily activities	0.282	0.044	0.322 ***		
	Social Support	0.058	0.025	0.109 *		

*Note.* PsyCap = psychological capital. GEC = Global Executive Composite. R^2^ indicates the variance explained by the overall model at each step, whereas ΔR^2^ indicates the additional variance explained by variables entered at that step. Significance markers associated with R^2^ refer to the overall model, whereas those associated with ΔR^2^ refer to the change in explained variance. * *p* < 0.05, ** *p* < 0.01, *** *p* < 0.001.

## Data Availability

The data presented in this study are available on request from the corresponding author.
